# The relationship between parental intimacy quality and young children’s emotional and behavioral problems: the chain mediating role of parenting stress and parent-child relationship

**DOI:** 10.3389/fpsyt.2025.1652814

**Published:** 2025-10-21

**Authors:** Ying Jiang, Jing Zhang, Konghui Shi, Xiaofen Wang

**Affiliations:** ^1^ Preschool Education Training and Research Department, Fujian Institute of Education, Fujian, China; ^2^ College of Education, Ningde Normal University, Fujian, China; ^3^ Chengyi College, Jimei University, Fujian, China

**Keywords:** parental intimacy quality, emotional and behavioral problems, parenting stress, parent-child relationship, young children

## Abstract

**Introduction:**

Young children’s emotional and behavioral problems represent a significant public health concern globally. This study investigated the underlying mechanisms of parental intimacy quality and young children’s emotional and behavioral problems, focusing on the roles of parenting stress and parent-child relationship. We hypothesized that: (1) Parenting stress plays a mediating role in the relationship between parental intimacy quality and young children’s emotional and behavioral problems; (2) Parent-child relationship plays a mediating role in the relationship between parental intimacy quality and young children’s emotions and problem behaviors; (3) Parenting stress and the parent-child relationship play a chain mediating role in the relationship between parental intimacy quality and young children’s emotional and behavioral problems.

**Method:**

An online survey was administered to 1868 young children’s parents in Fujian province, China, using convenience sampling methods. Data were analyzed using structural equation modeling (SEM) with the software Mplus 7.4 to examine the relationships between parental intimacy quality, parenting stress, parent-child relationship and young children’s emotional and behavioral problems.

**Results:**

The findings indicated that (1) parenting stress plays a mediating role in the relationship between parental intimacy quality and young children emotional and behavioral problems; (2) parent-child relationship plays a mediating role in the relationship between parental intimacy quality and young children emotional and behavioral problems; (3) parenting stress and parent-child relationship play a chain mediating role in the relationship between parental intimacy quality and young children’s emotional and behavioral problems.

**Conclusion:**

Our study highlights the close relationship between parental intimacy quality and young children’s emotional and behavioral problems, in which context parenting stress and parent-child relationship play a vital role.

## 1 Introduction

Ages 3 to 6 represent a period of rapid development in young children’s physical, cognitive, emotional, and social abilities, and it is also the stage most prone to psychological and behavioral deviations (1). According to statistics from the World Health Organization, about 10% to 20% of children worldwide suffer from at least one psychological problem (2). Among these, emotional and behavioral problems are more common in preschool-aged children (3).

A longitudinal study conducted in Australia involving 6 to 7-month-old infants and their families found that approximately 20% of young children exhibited emotional and behavioral problems (4). In different regions of China, the detection rate of emotional and behavioral problems among young children varies widely, ranging from 6% to 43.6% (5). In the short term, emotional and behavioral problems directly affect preschool children’s intelligence, cognition, and learning, leading to difficulties in making friends and growth retardation (6). In the long term, emotional and behavioral problems hinder social development and may increase the risk of a series of adverse outcomes later in life, such as mental disorders, antisocial behavior, substance abuse, and criminal activity (7).

Therefore, it is essential to examine the factors influencing children’s emotional and behavioral problems and their underlying mechanisms, so as to develop recommendations for enhancing the quality of their development.

### 1.1 The relationship between parental intimacy quality and emotional and behavioral problems in young children

Ecological systems theory asserts that the family is the microsystem exerting the greatest influence on individual development (8). Parental intimacy, as a component of the family ecosystem, is closely linked to the emotional and behavioral growth of young children. In this study, intimacy is narrowly defined as the marital relationship between the parents of young children. Emotional security theory posits that the quality of parental relationships affects children’s psychological development by shaping their perceived emotional security in the home environment (9). A harmonious parental relationship can reduce children’s emotional insecurity and foster secure attachment and positive emotional development. Conversely, a conflicted relationship can trigger children’s stress responses, leading to anxiety, aggression, and other behavioral issues (10). Furthermore, studies have shown that parental intimacy negatively predicts children’s aggression (11–13).

However, previous studies have focused primarily on the direct impact of single variables —such as parent-child relationships or parenting stress— on young children’s psychological development (14, 15). These studies overlook the interconnected roles of parental intimacy quality, parenting stress, and the parent-child relationship, failing to fully elucidate the underlying mechanisms through which parental intimacy quality influences children’s emotional and behavioral outcomes.

Therefore, the present study integrates parental intimacy quality, parent-child relationship, and young children’s development into a unified model to reveal the mechanism that link parental intimacy quality to young children’s emotional and behavioral problems, thereby providing an empirical foundation for more effective interventions.

### 1.2 Theoretical framework and research hypotheses

To systematically explain how family factors influence child development, this study integrates Bronfenbrenner’s ecological systems theory (8), the family stress model (16), and attachment theory (17) to form a coherent theoretical framework. Bronfenbrenner’s ecological systems theory provides the overarching multilevel perspective and identifies the family microsystem as the proximal context in which parental intimacy operates and shapes children’s development. Within this microsystem, the family stress model uniquely explains an affective-behavioral pathway: external pressures (e.g., interparental conflict) increase parental psychological distress, which undermines caregiving and thus justifies parenting stress as a mediator between parental intimacy quality and child outcomes. Attachment theory, by contrast, emphasizes how the emotional bond and internal working models formed in parent-child interactions determine children’s emotion regulation and social behavior, and thus uniquely justifies parent-child relationship quality as a mediator. Moreover, combining the family stress model and attachment theory suggests a plausible chain process—low parental intimacy may first elevate parenting stress, which then erodes parent-child relationship quality and in turn affects children—so that a chain mediation is theoretically warranted.

**Figure 1:**
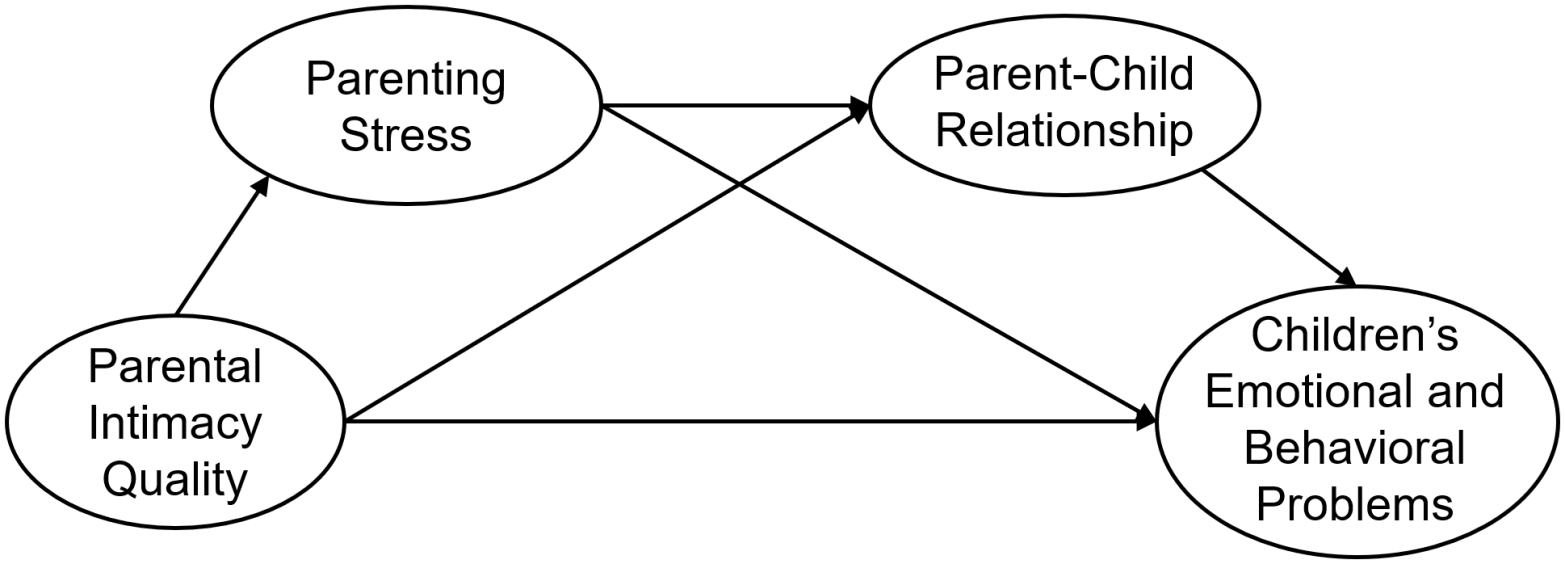
Chain mediated model hypothesis diagram.

In summary, we propose the following hypotheses, which are illustrated in the chain mediation model (see **Figure 1**):

H1: Parenting stress plays a mediating role in the relationship between parental intimacy quality and young children’s emotional and behavioral problems.
H2: Parent-child relationship plays a mediating role in the relationship between parental intimacy quality and young children’s emotions and problem behaviors.
H3: Parenting stress and the parent-child relationship play a chain mediating role in the relationship between parental intimacy quality and young children’s emotional and behavioral problems.

### 1.3 Evidence for the mediating role of parenting stress

Parenting stress refers to the pressure parents experience while fulfilling their parental roles and interacting with their children (16). On one hand, according to the family stress model (17), tense marital relationships cause parents to expend significant emotional resources due to conflict, thereby reducing the cognitive and emotional resources available for parenting. This reduction leads to decreased parenting efficacy and increased parenting stress (18). On the other hand, several cross-sectional studies have shown that parenting stress is significantly associated with young children’s internalizing problem behaviors (such as anxiety and depression) and externalizing problem behaviors (such as oppositional behavior, aggression, and hyperactivity) (19).

### 1.4 Evidence for the mediating role of parent-child relationship

Parent-child relationship refers to the interactive relationship between parents and children, encompassing both emotional communication and behavioral interaction. Family systems theory posits an influence between the marital subsystem and the parent-child subsystem, and the quality of the parent-child relationship directly shapes interaction patterns through emotional transmission (20). A harmonious and stable relationship between parents provides a growth environment rich in emotional support, which promotes healthy emotional and behavioral development in children (21). Secondly, attachment theory emphasizes the importance of the parent-child relationship in children’s early development (22). Without good parent-child relationship that provides emotional support and behavioral guidance, young children’s behavioral development can be adversely affected (22, 23).

### 1.5 Evidence for the chain mediating role of parenting stress and parent-child relationship

Many studies have examined the influence of parenting stress on the parent-child relationship. For example, parents who experience higher levels of parenting stress are more likely to encounter problems in their relationships with their children (19, 24). Elevated parenting stress diminishes parents’ emotional availability and sensitivity, which in turn harms the parent-child relationship.According to the stress process model (25), parenting stress caused by poor parental intimacy overflows into the realm of parent-child interaction, forming a chain of relationship deterioration: “parental intimacy quality → parenting stress → parent-child relationship.” In other words, the quality of intimacy serves as an environmental input that first influences parental psychological processes (parenting stress), which then alters parenting behaviors, affecting the parent-child relationship and ultimately child development.

### 1.6 The present study

In summary, this study will, grounded in ecosystem theory, the family stress model, and attachment theory, investigate the relationship between parental intimacy quality and their young children’s emotional and behavioral problems, as well as the mechanisms underlying this relationship. To address these questions, we collected data by distributing questionnaires to parents raising young children and examined the relationships among variables using structural equation modeling. This chain-mediating model enables us to identify more scientifically grounded parenting strategies and provides both theoretical and empirical support for promoting healthy development in young children.

## 2 Methods

### 2.1 Participants and measurement procedures

Between December 2024 and January 2025, data were collected using an online survey hosted on the Wenjuanxing platform (https://www.wjx.cn/). The research team prepared the questionnaire and generated an online completion link. Using the team’s personal and professional networks, we distributed the survey link and an investigator-prepared information sheet (detailing the study objectives and potential benefits for parents) to kindergarten principals. After reviewing the materials, the principals disseminated them to class teachers through the schools’ internal management systems. The class teachers then shared the survey link and information sheet in parent contact groups to recruit participants. Parents who were interested and met the eligibility criteria could access the link and complete the questionnaire themselves. The information sheet explicitly stated that only parents currently raising young children were eligible to participate.

Before proceeding to the formal questionnaire, all participants were presented with instructions clarifying that their participation was entirely voluntary and that their privacy would be protected. These instructions also reiterated the purpose of the study, emphasized the confidentiality and anonymity of responses, and informed participants of their right to withdraw from the survey at any time without penalty. Parents could begin the formal questionnaire only after reading the instructions and clicking the button labeled “I have read and agree to the above information.” This study was reviewed and approved by the Ethics Committee of Ningde Normal University. All procedures were conducted in accordance with governmental regulations and the 1964 Helsinki Declaration.

There were 2010 initial data entries received for this study. We then screened the collected data according to the following criterion: any respondent who selected the “agree” range on the 1–5 lie-detector scale for the item “Oranges are not fruits” was deemed to have provided an invalid response and was excluded from further analysis. After screening, the final valid dataset comprised 1868 entries, yielding an effective rate of 92.94%. Of these, 302 respondents were fathers (16.17%) and 1,566 were mothers (83.83%). Regarding parental age composition: 225 were under 30 years old (11.19%), 1,501 were between 30 and 40 years old (74.68%), 268 were between 41 and 50 years old (13.33%), and 16 were 50 years old or above (0.80%). In the corresponding group of young children (for families with more than one child, the oldest child attending preschool was surveyed), there were 932 boys (49.89%) and 936 girls (50.11%), with an average age of 4.76 ± 1.93 years.

### 2.2 Research tools

#### 2.2.1 Intimate relationship satisfaction questionnaire

Parental intimacy quality between parents was measured using the intimate relationship satisfaction questionnaire (26) developed by Shen (27). The questionnaire comprises seven items and employs a 5-point Likert scale ranging from 1 (not at all) to 5 (completely). Higher scores indicate greater quality with the couple’s intimate relationship. In this study, the Cronbach’s α coefficient for the questionnaire was 0.89.

#### 2.2.2 Parenting stress index-short form-15

Parenting stress was measured using the PSI-SF-15, revised by Luo, Wang (28). The PSI-SF-15 comprises three dimensions: parenting distress, dysfunctional parent-child interaction, and difficult child. The scale contains 15 items and employs a 5-point Likert scale ranging from 1 (not at all) to 5 (completely). Higher scores indicate greater parenting stress. In this study, the scale’s Cronbach’s α coefficient was 0.92.

#### 2.2.3 Parent-child relationship scale

The relationship between parents and young children was measured using the parent-child relationship scale, revised by Zhang, Chen (29). The scale comprises 22 items across two dimensions—intimacy and conflict (the latter reverse-scored)—with responses on a 5-point scale ranging from 1 (not at all) to 5 (completely). Higher total scores indicate a closer, more intimate relationship between the child and the parent. In this study, the Cronbach’s α coefficient for the scale was 0.77.

#### 2.2.4 Strengths and difficulties questionnaire (Chinese parent version)

Young children’s emotional and behavioral difficulties were assessed using the Difficulties scale of the strengths and difficulties questionnaire (Chinese parent version) (30), developed by Goodman (31). The Difficulties sub-questionnaire comprises four dimensions—emotional symptoms, conduct problems, hyperactivity, and peer relationship problems—and contains a total of 20 items. Responses are rated on a 3-point Likert scale ranging from 0 (does not apply) to 2 (applies fully). Higher total scores on the difficulties scale indicate more severe emotional and behavioral problems in young children. The Cronbach’s alpha for this questionnaire in the present study was 0.80.

### 2.3 Data Analysis

(1) Descriptive and correlational analyses of the variables were conducted using SPSS 20.

(2) Mplus 7.4 was used to test the relationship between parental intimacy quality and young children’s emotional and behavioral problems, with parenting stress and the parent-child relationship serving as chain mediators. Parameter estimation was performed using the MLM method, and mediating effects were tested using a bias-corrected, nonparametric, percentile bootstrap test. The bias-corrected, nonparametric, percentile bootstrap method provides accurate confidence intervals for the mediating effect of the product of the regression coefficients and can handle non-normal data; a mediating effect is considered significant if the 95% confidence interval for the estimated effect does not include zero (32).

## 3 Results

### 3.1 Common method bias test

To test whether there was a significant common method bias effect in this study, Harman’s one-way test was performed by constructing a one-way factor analysis using Mplus 7.4 with all questionnaire topics as indicators. The results revealed that the fit index of the model was *χ* ^2^/*df =* 13.89, RMSEA = 0.8, CFI = 0.52, SRMR = 0.8, which was a poor fit. Thus, there was no significant common method bias in this study.

### 3.2 Descriptive statistics of each variable

In order to test whether there is a close relationship between the variables and to prepare for the structural equation model, the Pearson correlation was used to test the relationship between the variables. The Pearson correlation analysis results are shown in **Table 1**. From **Table 1**, parental intimacy quality is significantly negatively correlated with parenting stress, significantly positively correlated with the parent-child relationship, and significantly negatively correlated with young children’s emotional and behavioral problems. Parenting stress is significantly negatively correlated with the parent-child relationship and significantly positively correlated with young children’s emotional and behavioral problems. The parent-child relationship is significantly negatively correlated with young children’s emotional and behavioral problems. These Pearson correlation results indicate that the main variables are closely related, so we can establish a structural equation model for further analysis. Additionally, because children’s age and gender are significantly correlated with the outcome variables, they will be included as control variables in the analytical model.

**Table 1:** Descriptive statistics of each variable and the results of their correlation analysis (*N* = 1868).

Variables	1	2	3	4	5	6
1 Age of young children	—					
2 Gender of young children ^a^	0.02	—				
3 parental intimacy quality	-0.03	-0.01	—			
4 parenting stress	0.06^*^	0.02	-0.488^***^	—		
5 parent–child relationship	-0.05^*^	-0.03	0.51^***^	-0.67^***^	—	
6 young children’s emotional and behavioral problems	0.02	-0.01	-0.43^***^	0.57^***^	-0.66^***^	—
*M*	4.76	0.49	4.11	2.17	3.97	1.50
*SD*	1.93	/	0.77	0.67	0.50	0.26

a Gender is a dummy coded variable, 0 = female, 1 = male, and the mean is the proportion of male young children; * indicates *p* < 0.05, *** indicates *p* < 0.001.

### 3.3 The mediating role of parenting stress and parent-child relationship

To test the three hypotheses proposed in this study, the chain mediating role of parenting stress and parent-child relationship in the relationship between parental intimacy quality and young children’s emotional and behavioral problems was examined by developing structural equation models using each questionnaire item packets or dimensions as an observational indicator. Given that the intimate relationship satisfaction questionnaire is unidimensional and contains a number of items, this study employed a balanced approach to parcel those items during the modeling process (33).

**Figure 2:**
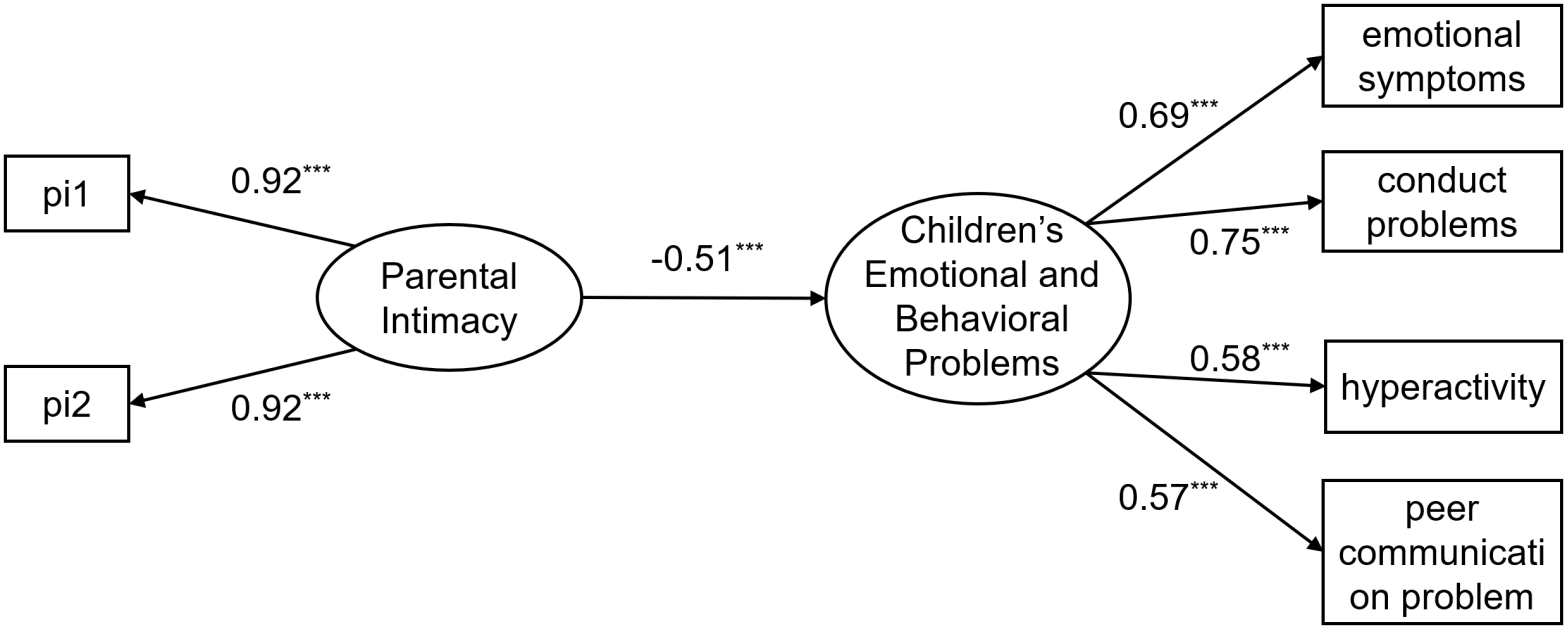
Direct effect model diagram. Figures presented in the figure are standardized solutions; pi1–pi2 are item parcels created using the parceling method for the intimate relationship satisfaction questionnaire; *** indicates *p* < 0.001.

First, the predictive effect of parental intimacy quality on young children’s emotional and behavioral problems was examined. The results indicate that, aside from the *χ*²/*df* value being inflated due to the sample size, the remaining indices suggest that the overall model fit is good: *χ* ^2^/*df =* 7.73, RMSEA = 0.06, CFI = 0.99, TLI = 0.97, SRMR = 0.02. The direct effects model is shown in **Figure 2**, according to which parental intimacy quality significantly and negatively predicted young children’s emotional and behavioral problems (*β* = -0.51, *p* < 0.001). These results suggest a robust relationship between the two, and mediating variables can be included to further analyze the mechanism through which parental intimacy quality relates to young children’s emotional and behavioral problems.

**Figure 3:**
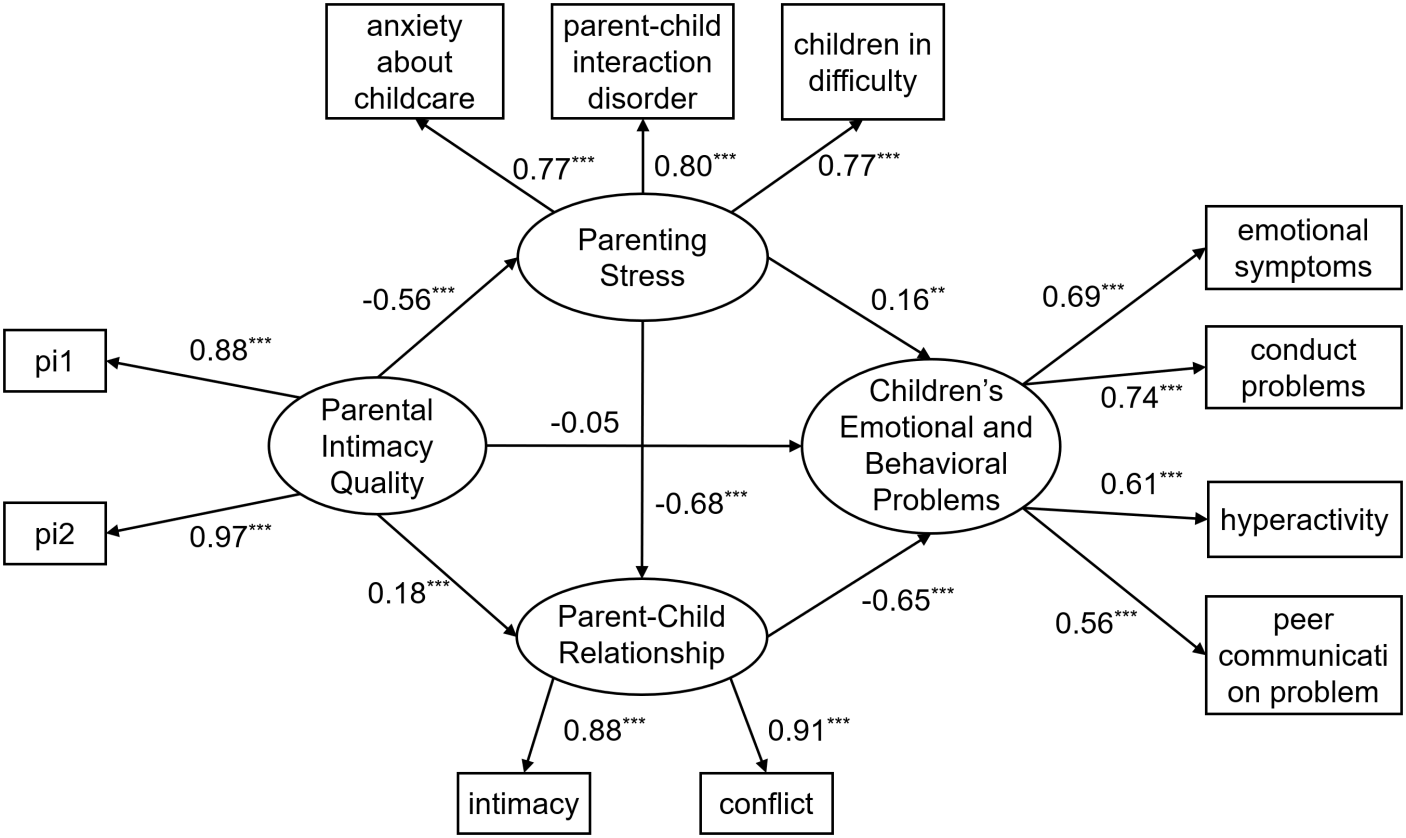
Diagram of chain mediated model. Figures presented in the figure are standardized solutions; pi1–pi2 are item parcels created using the parceling method for the intimate relationship satisfaction questionnaire; **p < 0.01, ***p < 0.001.

Next, the mediating role of parenting stress and parent-child relationship in the relationship between parental intimacy and young children’s emotional and behavioral problems was examined. The results indicate that, aside from the *χ*²/*df* value being inflated due to the sample size, the remaining indices suggest that the overall model fit is good: *χ* ^2^/*df =* 8.62, RMSEA = 0.06, CFI = 0.97, TLI = 0.96, SRMR = 0.03. The mediation model is shown in **Figure 3**. The model results indicated that parental intimacy quality significantly and negatively predicted parenting stress (*β* = -0.56, *p* < 0.001) and significantly and positively parent-child relationship (*β* = 0.18, *p* < 0.001). Parenting stress significantly and positively predicted young children’s emotional and behavioral problems (*β* = 0.16, *p* < 0.001) and significantly and negatively predicted parent-child relationship (*β* = -0.68, *p* < 0.001). Parent-child relationship significantly and negatively predicted young children’s emotional and behavioral problems (*β* = -0.68, *p* < 0.001). The results indicated that each pathway for this mediating influence was significant and could be used to examine the chain mediating effect of parenting stress and parent-child relationship on the relationship between parental intimacy quality and young children’s emotional and behavioral problems.

The bias-corrected nonparametric percentile bootstrap method (5000 replicate samples) was used to test whether the mediating effects of parenting stress and parent-child relationship were significant between parental intimacy quality and young children’s emotional and behavioral problems (see **Table 2** for results).

**Table 2:** Mediated effect values and bootstrap test results.

Paths	Effect value	Bootstrap confidence intervals
Total effect	-0.51^***^	[-0.55, -0.46]
parental intimacy quality→parenting stress→young children’s emotional and behavioral problems	-0.09^*^	[-0.16, -0.02]
parental intimacy quality→parent–child relationship→young children’s emotional and behavioral problems	-0.11^***^	[-0.16, -0.07]
parental intimacy quality→parenting stress→parent–child relationship→young children’s emotional and behavioral problems	-0.25^***^	[-0.30, -0.20]

The figures listed in the table are standard solutions. * *p* < 0.05, *** *p* < 0.001.

As shown in **Table 2**, the mediating effect of parenting stress between parental intimacy quality and young children’s emotional and behavioral problems was significant (mediating effect value of -0.09, *p* < 0.05, 95% CI = [-0.16, -0.02]). The mediating effect accounted for 17.65% of the total effect, confirming H1. The mediating effect of parent-child relationship between parental intimacy quality and young children’s emotional and behavioral problems was significant (mediating effect value of -0.11, *p* < 0.001, 95% CI = [-0.16, -0.07]). The mediating effect accounted for 21.57% of the total effect, confirming H2. The chain mediating effect of parenting stress and parent-child relationship between parental intimacy quality and young children’s emotional and behavioral problems was significant (mediating effect value of -0.25, *p* < 0.001, 95% CI = [-0.30, -0.20]). The mediating effect accounted for 49.02% of the total effect, confirming H3.

## 4 Discussion

Based on existing theoretical models and previous studies, this research constructed a chain mediation model to examine the parental marital relationship, parent-child relationship, and early childhood development within the same framework.

Data were collected using questionnaires and analyzed through correlation analysis and structural equation modeling to investigate the chain mediating roles of parenting stress and the parent-child relationship in the link between parental intimacy quality and young children’s emotional and behavioral problems.

The main findings revealed that parenting stress and the parent-child relationship serve as mediating variables in this relationship through three pathways: (1) The mediating role of parenting stress; (2) The mediating role of the parent-child relationship; (3) The chain mediating role of parenting stress and the parent-child relationship.

### 4.1 The mediating role of parenting stress

This study found that parenting stress plays a mediating role in the relationship between parental intimacy quality and young children’s emotional and behavioral problems; in other words, parental intimacy quality may influence young children’s emotional and behavioral problems through parenting stress.

First, the quality of parental intimacy may negatively affect parenting stress; the poorer the intimacy between parents, the greater the parenting stress, which aligns with the findings of previous studies (34). The family stress model (17) states that intrafamilial stress (e.g., parental relationship disharmony) creates an unstable family atmosphere, weakening parenting commitment and generating parenting stress. If the parental relationship is tense and conflicts are frequent, parents may expend more energy and emotional resources on couple conflicts, exhibit lower parenting sensitivity, and be more likely to shift responsibilities in the parenting process. This leads to a higher psychological burden on individual parents—such as prolonged anxiety and depression—which, in turn, exacerbates parenting stress (35, 36).

Second, parenting stress may positively affect young children’s emotional and behavioral problems: the higher the parenting stress, the more emotional issues and problematic behaviors appear in young children, which aligns with the results of previous studies (37–39). According to the stress spillover effect in the family stress model (36), parents who experience high levels of parenting stress over an extended period may become emotionally depleted, leading to negative responses toward their young children (e.g., yelling or indifference), frequent temper tantrums aimed at their children, and negative emotional expressions such as fear and anxiety. Over time, these parental responses may contribute to long-term behavioral problems in young children, such as withdrawal and aggression.

### 4.2 The mediating role of parent-child relationship

This study found that the parent-child relationship plays a mediating role in the relationship between parental intimacy quality and young children’s emotional and behavioral problems. In other words, parental intimacy quality may influence young children’s behavioral development through the parent-child relationship.

First, the quality of parental intimacy may have a positive effect on the parent-child relationship, which aligns with the results of previous studies (40, 41). Family systems theory states that the family is an interconnected system in which individual family members interact with and influence one another. If parental tension and conflict are constant, and the family atmosphere is characterized by hostility and repression, parents may project their negative emotions onto their children or neglect their children’s emotional needs, leading to problems in the parent-child relationship (42).

Second, the parent-child relationship may have a negative impact on young children’s emotional and behavioral development. The worse the parent-child relationship is, the more likely young children are to experience difficulties in their behavioral development, which is consistent with the findings of previous studies (43). Attachment theory suggests that secure attachment relationships are central to early childhood development (44). Young children who do not have a strong parent-child relationship with their parents are unlikely to develop secure attachment relationships. In this case, young children may not have sufficient close interaction with their parents, and they may not have sufficient opportunities to learn how to express their emotions and understand the feelings of others, thus increasing the risk of emotional and behavioral problems.

### 4.3 The chain mediating role of parenting stress and parent-child relationship

This study found that parenting stress and the parent-child relationship play a chain mediating role in the relationship between parental intimacy quality and young children’s emotional and behavioral problems. Specifically, when parental intimacy quality is low, parenting stress may increase, which in turn negatively affects the parent-child relationship, ultimately heightening the risk of emotional and behavioral problems in young children.

According to the family stress model (17), when parents are estranged or in conflict, this depletes psychological resources, reduces parenting sensitivity, and amplifies stress perceptions, leading to increased parenting stress. In turn, persistent parenting stress may cause parents to exhibit more negative parent-child interaction behaviors, such as a lack of patience with their children, over-criticism, and neglect of their children’s emotional needs. These negative behaviors can undermine the trust and intimacy between parents and children, leading to a strained parent-child relationship (22). When the parent-child relationship is poor, young children may feel insecure, neglected, or unloved, which makes them more prone to various emotional and behavioral problems (45).

In summary, the chain mediated model of this study verifies that family stress has a cascading transmission (46). This finding expands the focus of established research on a single mediated pathway, emphasizes the complexity of the multiple linkages within the family system, and enriches our understanding of the mechanisms by which the family environment influences early childhood development.

### 4.4 Suggestion

Based on the findings of the present study, we offer the following practice-oriented recommendations for community and school implementation:

1. Implement family-support programs that prioritize improvement of parental relationship quality. Given the significant influence of parental relationship quality on young children’s emotional and behavioral problems, community organizations and schools should proactively provide targeted interventions such as couple-communication skills workshops and seminars on marital relationships. Program content might include emotion recognition and regulation, nonviolent communication, conflict-resolution strategies, and joint co-parenting planning. Equipping parents with effective communication and conflict-management skills can help cultivate a harmonious family psychosocial environment at its source and thereby prevent the onset of emotional and behavioral problems in early childhood.
2. Implement a comprehensive, dual-target intervention aimed at both reducing parenting stress and enhancing parent-child interaction. In light of the mediating role of parenting stress and parent-child relationship quality, we recommend the development of a multi-tiered support system. For stress management, community and educational agencies should establish online and in-person parenting consultation platforms and parent peer-support groups to provide parents with immediate problem-solving assistance and opportunities for experience sharing, thereby creating sustainable sources of social support and stress-buffering mechanisms. For the parent-child relationship, schools and community organizations should systematically organize activities such as parent-child sports days and parent-child reading clubs. Activity design should explicitly operationalize elements of “high-quality parental engagement” (e.g., sensitive responsiveness, shared attention, contingent communication) and guide parents to increase high-quality time with their children, directly strengthening the parent-child emotional bond and promoting the emotional and behavioral development of young children.

### 4.5 Limitations and directions for future research

Several limitations of this study are noteworthy (1). The current study utilized a cross-sectional design, future research can utilize an intensive tracking design to elucidate the causal linkages between independent and dependent variables. Future research also can employ multi-informant approaches (e.g., teacher ratings, systematic classroom observations, and administrative records) alongside longitudinal designs to provide stronger evidence for the temporal ordering of effects and to strengthen causal inference. (2) This study used self-report measures for data collection, which made the findings less objective. In the future, it may be possible to increase the objectivity of the results by reaching out to parents and either using interviews or conducting observations to gather relevant information. (3) This study was conducted in China. Cultural differences across countries may produce different results. Future research can undertake cross-cultural validation of the relationships among the variables identified in the present study.

## 5 Conclusion

This study utilizes a questionnaire-based research approach to investigate the mechanisms underlying the relationships among parental intimacy quality, parenting stress, parent-child relationship, and young children’s emotional and behavioral problems. The study led to the following conclusions:

1. Parenting stress plays a mediating role in the relationship between parental intimacy quality and young children’s emotional and behavioral problems.
2. Parent-child relationship plays a mediating role in the relationship between parental intimacy quality and young children’s emotional and behavioral problems.
3. Parenting stress and parent-child relationship play a mediating role in the relationship between parental intimacy quality and young children’s emotional and behavioral problems.

## Data Availability

The raw data supporting the conclusions of this article will be made available by the authors, without undue reservation.

## References

[1] Racz SJ King KM Wu J Witkiewitz K McMahon RJ. The predictive utility of a brief kindergarten screening measure of child behavior problems. J consulting Clin Psychol. (2013) 81:588–99. doi: 10.1037/a0032366, PMID: 23544679 PMC3752994

[2] Bele SD Bodhare TN Valsangkar S Saraf A. An epidemiological study of emotional and behavioral disorders among children in an urban slum. Psychology Health Med. (2013) 18:223–32. doi: 10.1080/13548506.2012.701751, PMID: 22783928

[3] Wan G-B Wei Z He H-J He M-Y. Prevalence of emotional disorders in preschool children in Shenzhen. Chin J Child Health Care. (2011) 19:1077–9. doi: CNKI:SUN:ERTO.0.2011-12-007

[4] Bayer JK Hiscock H Ukoumunne OC Price A Wake M. Early childhood aetiology of mental health problems: a longitudinal population-based study. J Child Psychol Psychiatry. (2008) 49:1166–74. doi: 10.1111/j.1469-7610.2008.01943.x, PMID: 18665879

[5] Liu Y-Y. Study summarization on the psychological health of chinese young children aging from 0 to 6 years old. Stud Preschool Educ. (2009) 06:10–5. doi: CNKI:SUN:XQJY.0.2009-06-004

[6] Hu M Jin J Jin Y Bao F Liu B-Y Guo Y-G. Cross-sectional study on behavior problems and influential factors among preschool children in Huangpu district of Guangzhou. Chin J Evidence-Based Pediatrics. (2012) 7:11–8. doi: CNKI:SUN:XZEK.0.2012-01-005

[7] Augustyn MB Fulco CJ Agbeke D Henry KL. The joint development of externalizing and internalizing behaviors in black and Hispanic youth and the link to late adolescent substance use. Dev psychopathology. (2022) 34:1144–62. doi: 10.1017/S0954579420001881, PMID: 33517946 PMC8325714

[8] Bronfenbrenner U. Ecology of the family as a context for human development: Research perspectives. Dev Psychol. (1986) 22:723–42. doi: 10.1037/0012-1649.22.6.723

[9] Davies PT Cummings EM. Marital conflict and child adjustment: an emotional security hypothesis. psychol bulletin. (1994) 116:387–411. doi: 10.1037/0033-2909.116.3.387, PMID: 7809306

[10] Cummings EM Davies P. Children and marital conflict: The impact of family dispute and resolution. New York, NY: Guilford Press (1994).

[11] Cummings EM Davies PT. Marital conflict and children: An emotional security perspective. New York, NY: Guilford Press (2011).

[12] Hughes C Devine RT Mesman J Blair C. Parental well-being, couple relationship quality, and children's behavioral problems in the first 2 years of life. Dev psychopathology. (2020) 32:935–44. doi: 10.1017/S0954579419000804, PMID: 31339479

[13] Li S Ma X Zhang Y. Intergenerational transmission of aggression: A meta-analysis of relationship between interparental conflict and aggressive behavior of children and youth. Curr Psychol. (2023) 42:32008–23. doi: 10.1007/s12144-022-04219-z

[14] Hatakka E. Longitudinal associations between parental bonding and child preschool social-emotional problems. development. (2023) 53:371–99. doi: 10.1111/jcpp.13687, PMID: 40715671

[15] Ribas LH Montezano BB Nieves M Kampmann LB Jansen K. The role of parental stress on emotional and behavioral problems in offspring: a systematic review with meta-analysis. Jornal pediatria. (2024) 100:565–85. doi: 10.1016/j.jped.2024.02.003, PMID: 38636551 PMC11662746

[16] Abidin RR. The determinants of parenting behavior. J Clin Child Psychol. (1992) 21:407–12. doi: 10.1207/s15374424jccp2104_12

[17] Conger KJ Rueter MA Conger RD. “The role of economic pressure in the lives of parents and their adolescents: The family stress model”. In: *Negotiating adolescence in times of social change* . Cambridge: Cambridge University Press (2000). pp. 201–23.

[18] Newland LA. Family well-being, parenting, and child well-being: Pathways to healthy adjustment. Clin Psychol. (2015) 19:3–14. doi: 10.1111/cp.12059

[19] Neece CL Green SA Baker BL. Parenting stress and child behavior problems: A transactional relationship across time. Am J intellectual Dev disabilities. (2012) 117:48–66. doi: 10.1352/1944-7558-117.1.48, PMID: 22264112 PMC4861150

[20] Cox MJ Paley B. Families as systems. Annu Rev Psychol. (1997) 48:243–67. doi: 10.1146/annurev.psych.48.1.243, PMID: 9046561

[21] Cummings EM Schatz JN. Family conflict, emotional security, and child development: translating research findings into a prevention program for community families. Clin Child Family Psychol Review. (2012) 15:14–27. doi: 10.1007/s10567-012-0112-0, PMID: 22311087

[22] Bowlby J. Attachment and loss. New York: Basic Books (1969).

[23] Pinquart M. Associations of parenting dimensions and styles with externalizing problems of children and adolescents: An updated meta-analysis. Dev Psychol. (2017) 53:873–932. doi: 10.1037/dev0000295, PMID: 28459276

[24] Tokunaga A Iwanaga R Yamanishi Y Higashionna T Tanaka K Nakane H. Relationship between parenting stress and children's behavioral characteristics in Japan. Pediatr Int. (2019) 61:652–7. doi: 10.1111/ped.13876, PMID: 31044477

[25] Abidin RR. Parenting stress index manual. Odessa, FL: Psychological Assessment Resources (1995).

[26] Vaughn MJ Matyastik Baier ME. Reliability and validity of the relationship assessment scale. Am J Family Ther. (1999) 27:137–47. doi: 10.1080/019261899262023

[27] Shen L. The Effects of Personality Traits and Interaction Styles on Intimate Relationship Satisfaction. Bei Jing, BJ: Beijing Normal University (2005).

[28] Luo J Wang M-C Gao Y Zeng H Yang W Chen W. Refining the parenting stress index–short form (PSI-SF) in Chinese parents. Assessment. (2021) 28:551–66. doi: 10.1177/1073191119847757, PMID: 31072108

[29] Zhang X Chen H-C Zhang G-F. Children's relationships with mothers and teachers: linkages to proble mBehavior in their first preschool years. Acta Psychologica Sin. (2008) 40:418–26. doi: 10.3724/SP.J.1041.2008.00418

[30] Kou J-H Du Y-S Xia L-M. Reliability and validity of "children strengths and difficulties questionnaire" in Shanghai norm. Shanghai Arch Psychiatry. (2005), 25–8.

[31] Goodman R. The Strengths and Difficulties Questionnaire: a research note. J Child Psychol Psychiatry. (1997) 38:581–6. doi: 10.1111/j.1469-7610.1997.tb01545.x, PMID: 9255702

[32] Wen Z-L Ye B-J. Analyses of mediating effects: the development of methods and models advances in psychological science. Adv Psychol Sci. (2014) 22:731–45. doi: 10.3724/SP.J.1042.2014.00731

[33] Wu Y Wen Z-L. Item parceling strategies in structural equation modeling. Adv psychol Sci. (2011) 19:1859–67. doi: CNKI:SUN:XLXD.0.2011-12-017

[34] Liu Y Li H. The impact of coparenting on parenting stress: The mediating role of parent-child intimacy and parent-child conflict. psychol Behav Res. (2023) 23:321–30.

[35] Peng Y Guo C Zeng J Chen S. The impact of coparenting on parenting stress: The mediating role of parent-child intimacy and parent-child conflict. psychol Behav Res. (2023) 21:485–96. doi: CNKI:SUN:CLXW.0.2023-04-006

[36] Kuo PX Johnson VJ. Whose parenting stress is more vulnerable to marital dissatisfaction? A within-couple approach examining gender, cognitive reappraisal, and parental identity. Family Process. (2021) 60:1470–87. doi: 10.1111/famp.12642, PMID: 33704779

[37] He H-Y Yu M Ning M Cui X-C Jia L-Y Li R-Y. The role of mother-child relationship in the association between maternal parenting stress and emotional and behavioral problems in preschool children. Zhongguo dang dai er ke za zhi= Chin J Contemp pediatrics. (2023) 25:394–400., PMID: 37073845 10.7499/j.issn.1008-8830.2210053 PMC10120346

[38] Liu L Wang M. Parenting stress and harsh discipline in China: The moderating roles of marital satisfaction and parent gender. Child Abuse neglect. (2015) 43:73–82. doi: 10.1016/j.chiabu.2015.01.014, PMID: 25676108

[39] Wang S-M Yan S-Q Xie F-F Cai Z-L Gao G-P Weng T-T. Association of preschool children behavior and emotional problems with the parenting behavior of both parents. World J Clin Cases. (2024) 12:1084–93. doi: 10.12998/wjcc.v12.i6.1084, PMID: 38464916 PMC10921310

[40] Brown GL Schoppe-Sullivan SJ Mangelsdorf SC Neff C. Observed and reported supportive coparenting as predictors of infant–mother and infant–father attachment security. Early Child Dev Care. (2010) 180:121–37. doi: 10.1080/03004430903415015, PMID: 25983376 PMC4430853

[41] Li M Gong H Zhang H Chen Y Zhang C. Maternal adult attachment and mother-adolescent attachment: the chain mediating role of marital satisfaction and harsh parenting. Front Psychiatry. (2023) 14:1170137. doi: 10.3389/fpsyt.2023.1170137, PMID: 37409160 PMC10319057

[42] Ramos AM Shewark EA Fosco GM Shaw DS Reiss D Natsuaki MN. Reexamining the association between the interparental relationship and parent–child interactions: Incorporating heritable influences. Dev Psychol. (2022) 58:43–54. doi: 10.1037/dev0001278, PMID: 35073119 PMC8973458

[43] Chen F-M Yuan Y-C Wang Y Zhang C. Parental conflict and infant’s problem behavior: A moderated mediating model. Chin J Clin Psychol. (2018) 26:736–41+670. doi: 10.16128/j.cnki.1005-3611.2018.04.023

[44] Bowlby J. A secure base: Parent-child attachment and healthy human development. New York: Basic books (2008).

[45] Iwanski A Lichtenstein L Forster F Stadelmann C Bodenmann G Zimmermann P. A family systems perspective on attachment security and dependency to mother and father in preschool: differential and reciprocal effects on children’s emotional and behavioral problems. Brain Sci. (2023) 13:35. doi: 10.3390/brainsci13010035, PMID: 36672018 PMC9856694

[46] Cox MJ Paley B. Understanding families as systems. Curr Dir psychol Sci. (2003) 12:193–6. doi: 10.1111/1467-8721.01259

